# The role of sortilin in the “Glut4 Pathway”.

**DOI:** 10.1080/19420889.2017.1393592

**Published:** 2017-12-14

**Authors:** Konstantin V. Kandror

**Affiliations:** Boston University School of Medicine, Boston, MA

**Keywords:** GGA, glucose uptake, Glut4, retromer, sortilin

## Abstract

Sorting receptor, sortilin, is highly expressed in metabolically active tissues, such as brain, liver, skeletal muscle, and fat. Specifically in adipocytes, sortilin plays an important role in the “Glut4 pathway” by sorting the insulin-responsive glucose transporter, Glut4, in early endosomes and trans-Golgi network and re-routing the transporter from degradation to the recycling pathway.

One of the most important physiological functions of insulin is to promote postprandial blood glucose clearance. As glucose cannot penetrate through the plasma membrane, insulin mobilizes specific proteins called glucose transporters. The major insulin-responsive glucose transporter protein, Glut4, is expressed primarily in fat and skeletal muscle cells. As Glut4 is a very stable protein, insulin does not acutely regulate its expression levels or specific activity, but rather, its intracellular localization. Under basal conditions, Glut4 is largely excluded from the plasma membrane and is localized inside the cell in small insulin-responsive vesicles (IRVs) and trans-Golgi network (TGN) that is likely to represent the IRV “donor” compartment (Fig. 1). In addition to Glut4, the IRVs compartmentalize several other major component proteins, namely sortilin, IRAP (insulin regulated amino peptidase), and LRP1 (low density lipoprotein receptor-related protein 1). The role of the latter two proteins in the “Glut4 pathway” is currently unknown. Activation of the PI3 kinase-Akt2 signaling pathway by insulin leads to a rapid fusion of the IRVs with the plasma membrane and delivery of Glut4 to the cell surface causing a dramatic increase in the plasma membrane glucose permeability. All in all, this mechanism accounts for a 10–40 fold rise of glucose uptake into adipocytes and skeletal myocytes with a corresponding decrease in blood glucose levels.^1,2^ After insulin withdrawal, Glut4 is internalized into early/sorting endosomes and then – retrieved to TGN where the IRVs are re-formed.^2^

**Figure 1.**
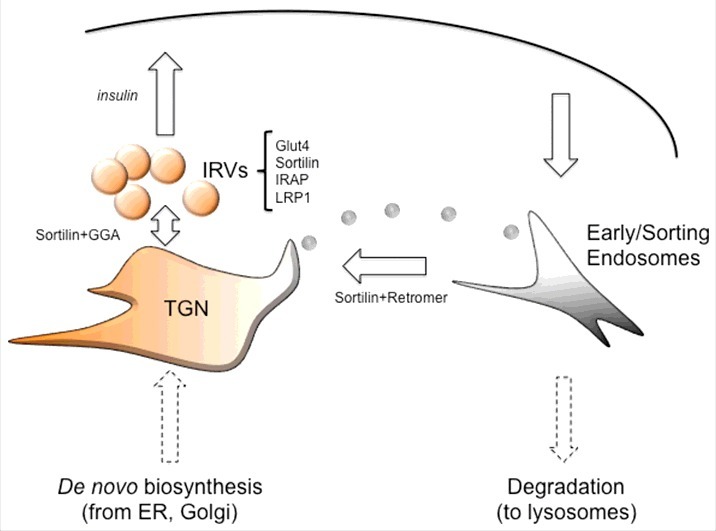
The “Glut4 pathway”. Under basal conditions, Glut4 is predominantly localized in small insulin-responsive vesicles (IRVs) that, in addition to Glut4, contain sortilin, IRAP, and LRP1. Formation of the IRVs on the donor membranes requires sortilin and GGA adaptors. Once formed, IRVs are retained intracellularly *via* an as yet unknown mechanism that may include the tethering protein TUG1. Upon insulin administration, the IRVs fuse with the plasma membrane. After that, Glut4 is internalized into early/sorting endosomes and retrieved to TGN *via* sortilin- and retromer-mediated mechanism.

There are at least two critical sorting steps in the Glut4 pathway: retrieval from early endosomes to the TGN, and formation of the IRVs on the TGN donor membranes (Fig. 1). Both these steps are enabled by the evolutionary conserved Vps10p family member, sortilin, which represents a type I transmembrane protein and a sorting receptor. Sortilin may act as a transmembrane scaffold protein: it binds to the first luminal/extracellular Glut4 in the lumen of endosomal membranes, and recruits retromer to the cytoplasmic side of the donor membrane *via* its C-terminus. This facilitates the distribution of Glut4 into vesicular carriers that translocate Glut4 from endosomes to TGN.^3^ Formation of the IRVs on TGN membranes may proceed *via* the same general mechanism; however, in this case, the C-terminus of sortilin interacts with GGA clathrin adaptors instead of retromer.^4,5^ Thus, sortilin together with retromer is required for the retrograde traffic of Glut4 from early endosomes to TGN, while sortilin and GGA may play the key role in the formation of the IRVs (Fig.1).

In line with this model, forced expression of Glut4 in “naïve” cells, such as undifferentiated fibroblasts that do not express sortilin but have normal amounts of retromer, leads to a rapid degradation of the transporter in lysosomes. However, double expression of sortilin and Glut4 increases the half-life of the latter and is sufficient to generate IRVs and to confer insulin-stimulated glucose transport to fibroblasts.^3,5^ Also, knock out of either sortilin or retromer in differentiated adipocytes re-routes Glut4 from TGN to lysosomes.^3^ In other words, both “gain of function” and “loss of function” experiments performed *in vitro* support the central role of sortilin in the Glut4 pathway.

Other experiments have shown that expression of sortilin in cultured cells, experimental animals, and humans is vulnerable to multiple factors that cause insulin resistance and diabetes, such as exposure to saturated fatty acids, high fat diet, obesity, and insulin resistance.^6–9^ At the same time, whole body sortilin knock out mice have decreased levels of glycolytic metabolites in adipose tissue but show no changes in Glut4 levels or in insulin-stimulated glucose uptake during insulin clamps.^10^ The difference between results obtained *in vitro* and *in vivo* may have several different explanations. Sortilin is expressed in multiple metabolically significant tissues, such as liver, brain, skeletal muscle, and fat. In each tissue sortilin participates in various processes exemplified by but not limited to signaling at the plasma membrane, protein targeting to lysosomes, retrograde traffic from endosomes to TGN, and secretion.^11^ Clearly, a complete genetic ablation of sortilin could change many biological variables with virtually unpredictable results which is reflected in complicated and often controversial phenotypes of transgenic and knock out animals.^10,12,13^ Vis-à-vis Glut4 expression levels and insulin-stimulated glucose uptake, it is possible that compensatory changes in Glut4 biosynthesis take place in response to increased Glut4 degradation that has not been addressed in the *in vivo* studies. It is also possible that in the absence of sortilin, Glut4 traffic *in vivo* can be assisted by other IRV proteins, such as IRAP or LRP1 that can also interact with Glut4, retromer, and clathrin adapters.^2^ In this regard, sortilin may not be the only protein responsible for the translocation of Glut4 between different membrane compartments. Rather, a web of interactions between various trafficking proteins and sorting receptors may provide a “safety net” for important cargo to reach its final destination in the cell.
